# Structured whole-body MRI highlights clinically relevant disease pattern changes in relapsed/refractory multiple myeloma

**DOI:** 10.1038/s41375-025-02834-w

**Published:** 2025-12-22

**Authors:** Martin Grözinger, Muriel Schlanke, Jana Gröne, Stella Erdmann, Marina Hajiyianni, Alexandra M. Poos, Heidi Thierjung, Elias K. Mai, Sandra Sauer, Dorothee Kaudewitz, Kaya Veelken, Christian S. Michel, Jan Hendrik Frenking, Lilli Sophie Sester, Tim Frederik Weber, Sam Sedaghat, Markus Wennmann, Carsten Müller-Tidow, Heinz-Peter Schlemmer, Stefan Delorme, Marc S. Raab, Hartmut Goldschmidt, Niels Weinhold, Lukas John

**Affiliations:** 1 German Cancer Research Center (DKFZ), Division of Radiology, Heidelberg, Germany; 2 Medical Faculty Heidelberg, University of Heidelberg, Heidelberg, Germany; 3 Institute of Medical Biometry, University of Heidelberg, Heidelberg, Germany, Heidelberg, Germany; 4 Heidelberg Myeloma Center, Department of Internal Medicine V, Heidelberg University Hospital, Medical Faculty Heidelberg, Heidelberg University, Heidelberg, Germany; 5 Clinical Cooperation Unit Molecular Hematology/Oncology, German Cancer Research Center (DKFZ), Heidelberg, Germany; 6 GMMG-Studygroup at Heidelberg University Hospital, Department of Internal Medicine V, Heidelberg University Hospital, Medical Faculty, Heidelberg University, Heidelberg, Germany; 7 Department of Internal Medicine V, Hematology, Oncology and Rheumatology, Heidelberg University Hospital, Medical Faculty, Heidelberg University, Heidelberg, Germany; 8 Clinic for Diagnostic and Interventional Radiology (DIR), Heidelberg University Hospital, Heidelberg, Germany

**Keywords:** Translational research, Myeloma

## Abstract

Whole-body imaging plays a critical role in assessing multiple myeloma (MM). The structured scoring systems MY-RADS and KIM score have primarily been developed for newly diagnosed patients (NDMM). However, their application and prognostic significance in relapsed/refractory multiple myeloma (RRMM) remains uncertain. To clarify this, we evaluated whole body magnetic resonance imaging (MRI) data from 46 RRMM patients and compared findings to 68 NDMM patients from the GMMG-HD7 trial using both scoring systems. Despite similar overall disease burden, RRMM patients showed significant differences, characterized by increased paramedullary and extramedullary disease and reduced diffuse marrow infiltration compared to NDMM. Both MY-RADS and KIM scores independently correlated with progression-free and overall survival in RRMM. These results highlight distinct biological patterns in RRMM, emphasizing a shift towards bone marrow-independent growth. Our findings suggest that in RRMM, iliac crest biopsies may underestimate disease burden, underlining the importance of imaging complementing bone marrow diagnostics.

## Introduction

Multiple myeloma (MM) is a plasma cell malignancy characterized by the accumulation of monoclonal plasma cells in the bone marrow, leading to destructive bone lesions, anemia, hypercalcemia, and renal dysfunction [1]. Despite advances in treatment, most MM patients eventually relapse and become refractory to therapy. Relapsed/refractory MM (RRMM) is characterized by a more aggressive disease biology, including increased genomic instability and clonal evolution [2, 3].

Whole-body magnetic resonance imaging (WB-MRI) has emerged as a powerful tool for assessing disease burden in MM due to its ability to detect both focal lesions and diffuse infiltration [4]. To standardize reporting of WB-MRI findings, the MY-RADS and KIM scoring systems have recently been developed [5, 6]. These scores have shown prognostic value in newly diagnosed MM (NDMM) patients, with higher scores associated with worse response [7]. However, the utility of the MY-RADS and KIM scoring systems in RRMM remains largely unknown.

With the hypothesis that RRMM patients exhibit a distinct pattern of diffuse and focal disease compared to NDMM patients, we conducted a prospective study to systematically assess the disease distribution in RRMM patients from our institution using MY-RADS and KIM scores and included 68 NDMM patients with identical imaging protocols for comparison. We show that RRMM exhibits more para- and extramedullary disease and significantly less diffuse disease than NDMM. We also demonstrate that the scores of structured radiological reporting systems impact progression-free (PFS) and overall survival (OS) independently of the presence of extramedullary disease. In conclusion, our data has implications for bone marrow-based diagnostics and prognosis of RRMM.

## Material and methods

### Patient cohort

The cohort comprised 47 RRMM patients, of which 46 had evaluable MRIs. All examinations took place at the German Cancer Research Center and Heidelberg University Hospital between August 2021 and January 2024. All examinations were performed before initiation of induction or relapse therapy. RRMM patients included in this study were required to have refractory or progressive disease assessed according to the IMWG criteria [8] with at least 2 prior lines of treatment. Presence of secondary malignancies was an exclusion criterion. The individual treatment regimens were selected based on physician’s choice and are listed in Supplementary Table 1. Follow up on RRMM patients was performed until December 2024 with a median followup of 18.3 months.

As a control group for comparing the overall distribution and imaging scores of disease burden, we included 82 NDMM patients, who were investigated using the same imaging protocol as RRMM patients on identical scanners between August 2018 and August 2021 of which 68 MRIs were suitable for evaluation. NDMM patient data originated from the GMMG HD7 clinical trial (ClinicalTrials.gov identifier: NCT03617731) [9]. Due to predefined study protocols and ongoing clinical follow-up within the HD7 trial framework, survival outcomes were not analysed for the NDMM cohort in this study.

**Fig. 1:**
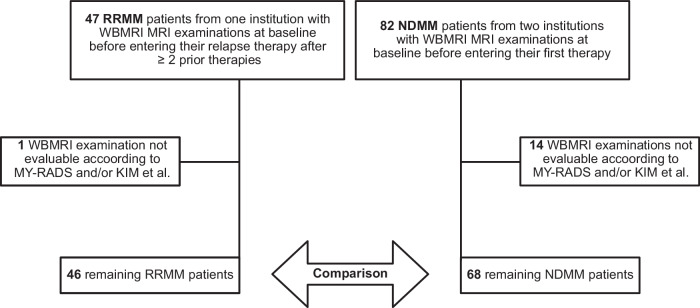
CONCEPT-diagram of included patients.

Relevant clinical parameters of both groups were collected prior to treatment, and bone marrow (BM) plasma cell infiltration (PCI) was determined as the maximum of both BM aspirate and BM trephine biopsy in line with International Myeloma Working Group (IMWG) diagnostic criteria. An overview of the included patients and their clinical characteristics is shown in Table 1 and Fig. 1.

**Table 1:** Clinical characteristics of NDMM and RRMM patients at initial diagnosis.

	NDMM patients (*N* = 68)	RRMM patients at initial diagnosis (*N* = 46)	RRMM patients (*N* = 46)
Characteristics	Median [Range]	Median [Range]	Median [Range]
Age [years]	59 [41–70]	56 [33–74]**	63 [37–78]
BM plasma cell infiltration [%]	55% [4–100%]	50% [5–95%]	13% [0–92%]***
Myeloma protein [g/l]	30.7 [0–78.7]	25.3 [0–87.2]	13.4 [0–83.7]***
SFLC-ratio	64.9 [1.0–10546.9]	51.4 [1.9–17500]	21.5 [1.2–4078.5]
Calcium [mmol/l]	2.4 [2.1–2.8]	2.4 [2.0–4.2]	2.3 [2.0–2.9]
Hemoglobin [g/dL]	11.9 [7.6–17.1]	12 [6.9–15.5]	11.2 [6.0–14.9]
Creatinine [mg/dL]	0.86 [0.56–2.32]	0.87 [0.57–9.5]	0.97 [0.55–2.39]**

Asterisks denote levels of statistical significance compared to the NDMM cohort using Wilcoxon rank sum test (*p < 0.05, **p < 0.01, ***p < 0.001).

### Image acquisition and protocol

Patients were examined using two 1.5 T Siemens MRI scanners (Siemens Magnetom Aera or Siemens Avantofit). The standardized non-contrast WB-MRI study protocol included coronary T1 turbo spin echo (TSE), coronary T2 TSE short tau inversion recovery (STIR), and axial diffusion weighted imaging (DWI) at b-values 50 (b50) and 800 (b800) (see Supplementary Table 2 for imaging parameters). Of note, since fat fraction was not part of the protocol for initial patients, it was not included for later patients to maintain consistency across all patients.

Both NDMM and RRMM patients were evaluated according to the same procedure. Initially, scans were analysed by a radiology resident and a board-certified radiologist within the clinical routines of our radiology departments. For the specific purpose of this study, all scans were then again analysed using MY-RADS and KIM scores by a third radiologist (MG) with 6 years of experience and a doctoral researcher (JG for NDMM patients and MS for RRMM patients) in a consensus read. The same radiologist (MG) participated in all readings. In case of uncertainty or disagreement, a fourth radiologist (SDE) with 35 years of experience provided final evaluation. SDE also approved the final results. Radiologists were blinded to clinical outcomes.

**Fig. 2:**
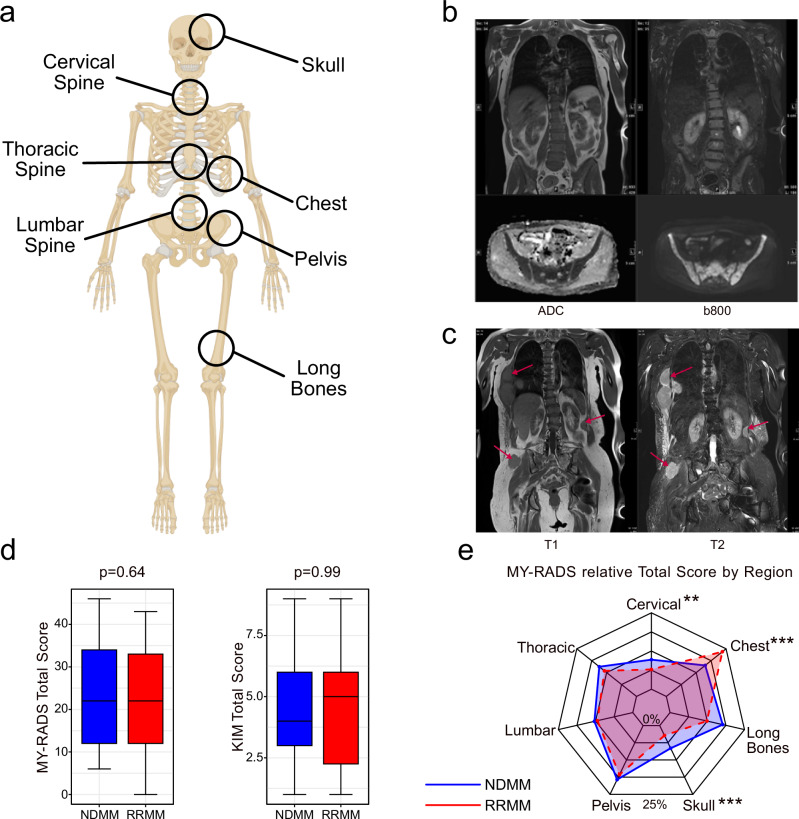
Different disease distribution patterns in NDMM and RRMM. **a** Visual depiction of the seven body regions assessed in MY-RADS-subscores. **b** Example of homogeneous diffuse marrow infiltration of the spine and pelvis in a NDMM patient. (ADC: Apparent diffusion coefficient; b800: b800 DWI sequence). **c** Example of multiple extra- and paramedullary manifestations in the chest of an RRMM patient. **d** Comparison of Total Scores according to KIM- and MY-RADS. No statistically significant difference was observed. **e** Radar chart showing the mean relative contribution to MY-RADS Total subscore by body region. Asterisks denote levels of statistical significance (*p < 0.05, **p < 0.01, ***p < 0.001).

The semiquantitative assessment based on the originally more descriptive structured reporting system MY-RADS was performed first. Each patient was analysed separately for seven body regions: cervical spine, thoracic spine, lumbar spine, pelvis, long bones, skull and the bony chest (Fig. 2a). Four subscores, namely for diffuse disease, the number of focal lesions, the size of the biggest lesions, and the occurrence of extra- or paramedullary lesions were manually evaluated for each body region and the sum of them formed the total score per skeletal region (ranging from 0 to 8 on an ordinal scale) (Fig. 2b, c). All seven skeletal regions together formed the total score for the entire patient (ranging from 0 to 56 on an ordinal scale). To better manage the disease burden caused by focal lesions in general, an additional score for the entire tumor burden adjusted by focal lesions was created, consisting of the sum of the two subscores for the number of focal lesions and the size of the biggest lesions. Instructions for calculating the total score are shown in Supplementary Table 3.

For the MY-RADS based analysis, a focal lesion was defined as an active myeloma lesion visible on all three sequences, hypointense on T1, mildly hyperintense on T2, and with characteristic apparent diffusion coefficient (ADC) values between 700 and 1400 µm²/sec with a diameter > 5 mm on the coronal T1-weighted sequence. Inactive lesions characterized by ADC-values > 1400 µm²/sec were not considered for the score [5, 10]. Diffuse bone marrow infiltration was categorized as 0 if mostly absent, or 1 if predominantly present. For assessing the disease burden from extra- and paramedullary manifestations, the MY-RADS score was used for each body region. Extramedullary disease was assigned to the spatially closest skeletal region.

The KIM score was not allocated separately for body regions [6]. A focal lesion was defined as visible on all three sequences with a diameter >10 mm on the coronal T1-weighted sequence. Diffuse bone marrow infiltration was assessed on a scale from 0 to 4, focusing primarily on axial skeleton involvement, especially the spine, with distinct infiltration patterns: Fat predominant (0 points), Variegated (1 point), Heterogeneous (2 points), Micronodular (3 points), Diffuse (4 points).

Extra- and paramedullary lesions were collectively evaluated in a single score with a scale from 0 to 2, 1 was assigned if only one lesion was present, and 2 if multiple lesions were present within the whole body.

### Statistical analysis

Followup for patients was conducted until December 2024. PFS was calculated as time from initiation of therapy until death or progressive disease, OS was calculated as time from study enrollment to death or last follow up. Spearman correlation was employed for correlation analyses, Wilcoxon rank sum test was employed to analyse differences between NDMM and RRMM for continuous variables, while chi-square test was used for binary variables. Group differences were examined through ordinal regression analysis. Survival was assessed using Kaplan-Meier curves and logrank test, with Cox-regression used for multifactorial survival analysis. Statistical significance was considered for *p* values < 0.05. As this is an exploratory study, no correction for multiple testing was performed. The statistical analysis was conducted using R (version 4.4.2).

### Writing process

During the preparation of this work the authors used OpenAI GPT-4.5 in order to improve clarity, readability, and coherence of some manuscript sections. After using this tool, the authors reviewed and edited the content as needed and take full responsibility for the content of the published article.

## Results

### Clinical characteristics

MRI data were available for 47 RRMM and 82 NDMM patients (Fig. 1). After excluding patients with missing sequences or metallic implants impeding MRI analysis, 46 RRMM and 68 NDMM patients were included in the analysis. Demographic data, laboratory measurements, and risk status are shown in Tables 1 and 2. Overall, clinical characteristics and laboratory measures were largely comparable between the cohorts, with the exception of RRMM patients being slightly younger at initial diagnosis (56 vs. 63 years, *p* = 0.003). The prevalence of high-risk cytogenetics and distribution of ISS and R-ISS at initial diagnosis was not significantly different between the groups.

**Table 2:** Clinical characteristics of NDMM and RRMM patients at initial diagnosis.

Characteristics at initial diagnosis	NDMM	RRMM
Sex [m:f]	46:22 [68% : 32%]	28:18 [61% : 39%]
ISS stage I [n, %]	40 [59%]	22 [54%]
ISS stage II [n, %]	16 [24%]	8 [19%]
ISS stage III [n, %]	12 [18%]	11 [27%]
R-ISS stage I [n, %]	29 [45%]	13[34%]
R-ISS stage II [n, %]	30 [47%]	18 [47%]
R-ISS stage III [n, %]	5 [8%]	7 [19%]
High risk del(17p), t(4;14), t(14;16) [n]	17[27%]	17 [40%]

For Cytogenetics, ISS and R-ISS, only patients with fully available data were considered. No statistically significant differences between the groups were observed.

### Comparable total disease burden but different distribution

First, we investigated the total MRI disease burden, which was quantified using both MY-RADS and KIM scores. We also evaluated contributions from individual skeletal regions (cervical spine, thoracic spine, lumbar spine, pelvis, long bones, skull, and chest) assessed in the MY-RADS score. MY-RADS and KIM scores were strongly correlated across all subscores and for the total score (R = 0.60–0.98, all *p* < 0.001; Supplementary Fig. 1). No statistically significant difference in total disease burden assessed by either scoring system was observed between RRMM and NDMM patients in our collective (mean MY-RADS score 22.1 vs. 23.7 in NDMM, *p* = 0.64; mean KIM score 4.6 vs. 4.5, *p* = 0.99, Fig. 2d, e). Region-by-region analyses revealed only subtle differences, with the skull and the cervical spine contributing slightly more to disease load in NDMM, albeit with minor absolute differences (skull: 8.2% vs. 4.1%, *p* < 0.001, Cervical spine 9.7% vs. 7.2%, *p* < 0.01). In contrast, the chest contributed more to overall disease load in RRMM patients (26.3% vs. 16.2%, *p* < 0.001) (Fig. 2e). Together, while tumor burden was similar between NDMM and RRMM, our data reveal distinct shifts in disease distribution across skeletal regions.

### Diffuse infiltration is significantly reduced in RRMM

**Fig. 3:**
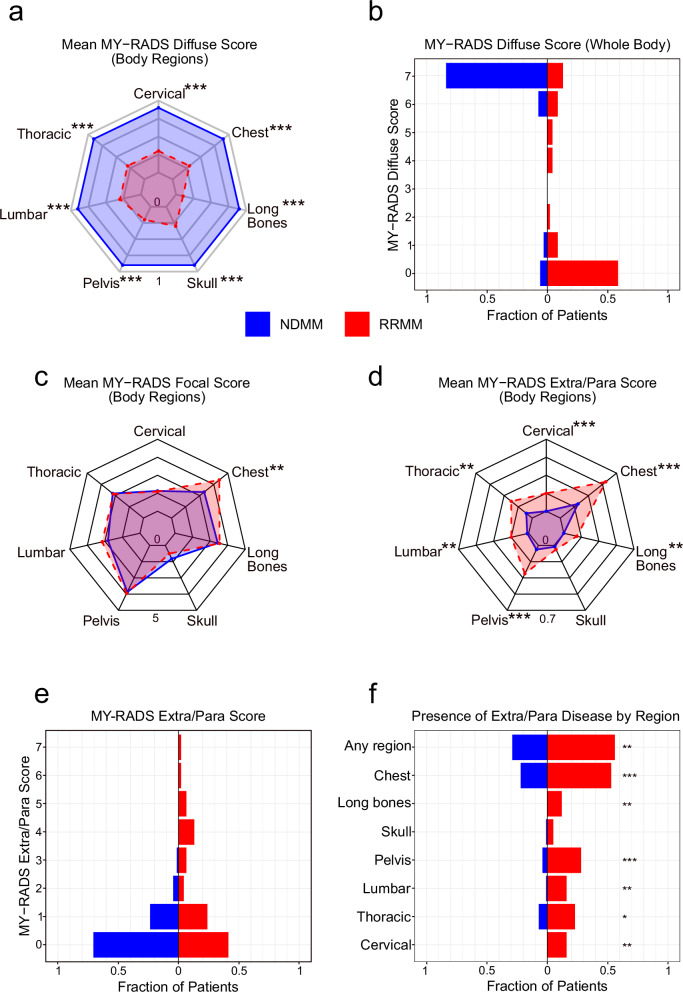
Comparative visualization of diffuse and extramedullary disease patterns in NDMM versus RRMM. **a** Radar chart showing the mean absolute MY-RADS diffuse subscore by body region (cervical spine, thoracic spine, lumbar spine, pelvis, skull, long bones, and chest) in NDMM (blue) vs. RRMM (red). **b** Whole-body MY-RADS diffuse scores for NDMM (blue) and RRMM (red), plotted as the fraction of patients in each score category. **c** Radar chart showing the mean absolute MY-RADS Focal subscore by body region. **d** Radar chart showing the mean absolute MY-RADS Extra/Para subscore by body region. **e** Whole-body MY-RADS Extra/Para scores by fraction of patients in NDMM. **f** Proportion of NDMM vs. RRMM patients exhibiting extra- and/or paramedullary disease in each body region, plus the overall incidence (“Any region”). Asterisks denote levels of statistical significance (*p < 0.05, **p < 0.01, ***p < 0.001).

We next analysed diffuse marrow infiltration using MY-RADS and KIM diffuse subscores across all skeletal regions. RRMM exhibited a strikingly lower diffuse score compared to NDMM (mean MY-RADS 2.0 vs. 6.3, *p* < 0.001; mean KIM 1.7 vs. 2.4, p < 0.001). Diffuse disease in any body region according to MY-RADS was not only significantly less common in RRMM overall (41% vs 91% in NDMM, *p* < 0.001) but also showed regional differences (Fig. 3a, b). While diffuse disease was prevalent throughout the skeleton in NDMM, it was notably rare in RRMM, particularly in the pelvis, where only 22% of RRMM patients showed diffuse disease compared to 91% of NDMM patients (*p* < 0.001), consistent with the lower median bone marrow plasma cell count at the iliac crest (Table 1). This finding underscores the risk of relying solely on iliac crest biopsies for disease assessment in relapsed patients.

### Extra- and paramedullary disease is markedly more common in RRMM

Given the decline in diffuse infiltration, we next examined whether RRMM patients show a compensatory increase in focal, para- or extramedullary lesions. Focal lesion burden was quantified using the MY-RADS Focal and KIM Focal subscores, while extra/paramedullary involvement was assessed separately using the respective subscores. Overall, the focal lesion burden was similar between NDMM and RRMM (mean MY-RADS Focal Score 17.0 vs. 18.5; *p* = 0.46, mean KIM Focal Score 1.7 vs. 1.9 *p* = 0.25). However, RRMM patients showed significantly higher scores in the chest (4.2 vs. 2.9, *p* = 0.001). Of note, the Extra/Para Score was significantly different, with RRMM showing significantly higher values across both scoring systems (mean MY-RADS Extra/Para Score 1.7 vs. 0.4, *p* < 0.001, mean KIM Extra/Para Score 0.96 vs. 0.41, p < 0.001). The prevalence of extra-/paramedullary disease in RRMM was almost twice that of NDMM (59% vs. 29%, *p* < 0.01), with significantly higher incidence and extent of extra- and paramedullary involvement in all skeletal regions, except the skull (Fig. 3d–f). Together, these results highlight a distinct shift in RRMM from diffuse infiltration to a pronounced preference for para- and extramedullary growth.

### Focal and extramedullary lesions drive prognosis in RRMM

**Fig. 4:**
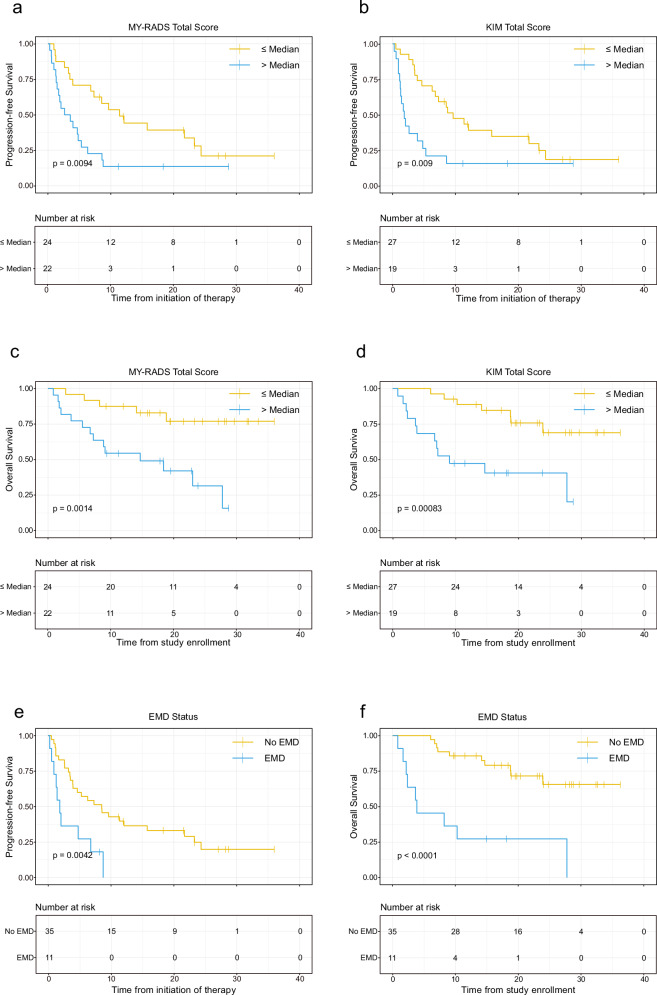
Kaplan-Meier survival curves illustrating progression-free survival (PFS) and overall survival (OS) in RRMM patients. Panels **a** and **b** show PFS stratified by median total scores of the MY-RADS and KIM scoring systems, respectively. Panels **c** and **d** depict OS using the same stratification. Panels **e** and **f** illustrate PFS and OS, respectively, stratified by the presence or absence of extramedullary disease (EMD). Survival probability is indicated on the y-axis, and time in months since initiation of therapy on the x-axis. *P* values are calculated using the log-rank test. Numbers at risk at each time point are provided beneath each curve.

Having established that RRMM patients have less diffuse infiltration but more bone marrow independent lesions, we next addressed the prognostic impact of the two total scores and respective subscores. Therefore, we first split RRMM patients at the median for total MY-RADS and KIM scores. To rule out treatment effects, we performed a subgroup comparison by treatment modality with no significant differences in MY-RADS or KIM scores observed between those treated with CAR-T cells (*n* = 14) and those receiving other regimens (Supplementary Tables 4 and 5). Across all patients, both higher MY-RADS Total Score and KIM Total Score were significantly associated with shorter PFS (*p* < 0.01, Fig. 4a, b). Similar results were seen for overall survival (OS) (Fig. 4c, d). Further breaking down scores into diffuse, focal and para/extramedullary components revealed that Focal Score and Extra/Para Score were associated with poor prognosis (Supplementary Fig. 2). In line with recent observations [11–13], dismal outcome was seen in patients with extramedullary disease, with a median PFS and OS of only 1.87 months and 3.87 months, respectively (Fig. 4e, f).

**Fig. 5:**
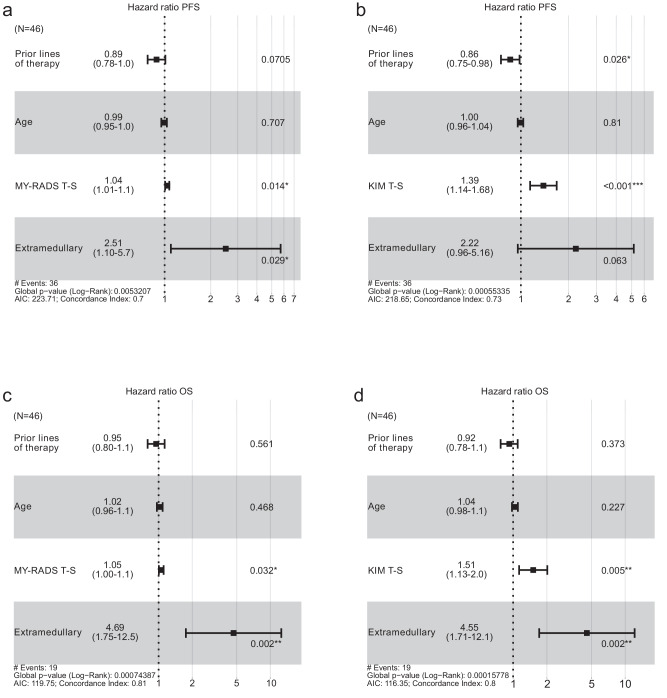
Cox proportional hazards models for 46 relapsed/refractory myeloma patients. **a** PFS model including MY-RADS Total score (T-S). **b** PFS model including KIM Total score (T-S). **c** OS model including MY-RADS total score (T-S). **d** OS model including KIM Total score (T-S). Hazard ratios (HR) with 95% confidence intervals appear for each covariate. The dashed vertical line at HR = 1.0 indicates no effect. Asterisks denote significance (*p < 0.05, **p < 0.01, ***p < 0.001).

As extramedullary disease (EMD) strongly influenced outcome, we conducted a multivariate analysis to determine whether total MY-RADS and KIM scores retained prognostic significance when EMD was taken into account. We included prior lines of therapy and patient age as covariates. The impact of MY-RADS Total Score on PFS (HR 1.04) was independent of EMD (*p* = 0.014). The same holds true for KIM Total Score (HR 1.39, *p* < 0.001). In both models, EMD was the strongest risk factor (HR 2.51 and 2.22, respectively, albeit only borderline statistically significant in the KIM-model) (Fig. 5a, b). Notably, in the model with KIM Total Score, an increased number of prior therapy lines appeared paradoxically protective—likely due to a “selection effect” favoring patients who tolerate multiple lines of therapy—rather than reflecting a genuine survival advantage. For OS (Fig. 5c, d), EMD consistently emerged as the strongest risk factor (HR 4.69/4.55, p = 0.002), but higher MY-RADS Total Score (HR 1.05, *p *= 0.03) and KIM Total Score (HR 1.51, *p* = 0.005) still contributed independently to worse OS. To further dissect which specific imaging features were driving these associations, we extended our analysis to a more granular level by evaluating individual MY-RADS and KIM subscores in multivariate Cox regression models. The Focal Score subscore of both MY-RADS (HR 1.04, *p* = 0.048) and KIM (HR 1.57, *p* = 0.017) was found to be independently associated with PFS (Supplementary Fig. 3). However, Focal Score was not associated with OS for either scoring system. Instead, in the KIM-model, the diffuse disease subscore emerged as an independent predictor of OS (HR 1.8, *p* = 0.01).

## Discussion

In our study, we systematically compared imaging patterns in RRMM and NDMM patients using structured whole-body MRI scoring systems (MY-RADS and KIM). Despite comparable total disease burdens between these patient groups, our findings reveal significant qualitative differences in disease distribution, with important implications for diagnosis, prognosis, and clinical management.

Firstly, we observed a pronounced reduction in diffuse bone marrow infiltration among RRMM patients compared to NDMM patients. This notable shift was most evident in regions traditionally assessed for marrow infiltration, such as the pelvis, where diffuse infiltration was significantly less frequent in RRMM. Consequently, this indicates a potential pitfall for the assessment of risk stratification markers such as cytogenetics and minimal residual disease (MRD) assessments relying solely on iliac crest biopsies. Such biopsies might underestimate true disease burden, particularly in patients who develop focal or extramedullary lesions elsewhere [14–16]. Given the advent of highly effective treatments, such as CAR-T cells and bispecific T-cell engagers, accurate assessment of MRD has become crucial [17–19]. Our findings therefore highlight the importance of incorporating comprehensive imaging approaches into standard diagnostic protocols to avoid false-negative MRD evaluations and to derive risk stratification markers without relying on tumor samples.

Secondly, our analysis demonstrated a marked increase in para- and extramedullary disease manifestations in RRMM patients. This shift toward bone marrow-independent growth underscores the aggressive biology characteristic of advanced myeloma [20–23]. The previously described prognostic relevance of extramedullary disease [11–13] was strongly confirmed in our study, where its presence significantly correlated with poorer progression-free and overall survival outcomes. Hence, the presence of extramedullary disease should be viewed as a critical high-risk feature, mandating intensified clinical surveillance and potentially more aggressive or targeted therapeutic interventions.

Thirdly, we validated the continued applicability of MY-RADS and KIM scoring systems, which were initially designed for NDMM, in the RRMM setting. Both scores retained independent prognostic relevance, predicting progression-free and overall survival even after accounting for extramedullary disease. Interestingly, however, the subscores associated with focal lesions predominantly predicted progression-free survival rather than overall survival. Furthermore, the KIM Diffuse subscore, unlike its MY-RADS counterpart, significantly correlated with overall survival, suggesting subtle, yet clinically meaningful differences between these scoring systems. While both scores correlated strongly, the subtle discrepancies observed underscore that WB-MRI scoring systems are inherently reductionist approaches to complex, high-dimensional imaging data, each carrying specific trade-offs. MY-RADS offers a more granular regional assessment and thus retains spatial information, but this comes at the cost of substantially longer evaluation time, limiting its feasibility in clinical routine. In contrast, KIM focuses on disease patterns and can be applied more feasibly alongside routine MRI reporting, although it sacrifices detailed spatial resolution. Given that EMD was an important predictor of PFS and OS and is not separately assessed in both scores, future iterations of these scores might benefit from recalibrating the weighting of extra- and paramedullary disease to reflect its heightened prognostic impact in RRMM patients.

Notably, while our study focused on RRMM, the prognostic impact of structured WB-MRI scoring in NDMM on PFS and OS remains an important open question. As survival in NDMM is considerably longer, meaningful PFS/OS analyses require longer follow-up. Future dedicated analyses in large, homogeneously treated NDMM cohorts, such as the GMMG HD7 trial, will be essential to establish the prognostic relevance of imaging scores at diagnosis and to complement bone marrow-based risk stratification in this setting.

The major limitation of this study is the relatively modest sample size and treatment heterogeneity in the RRMM setting, which limited the power for subgroup analyses. Although exploratory comparisons between patients treated with CAR-T cells and those receiving other regimens revealed no significant differences in MY-RADS or KIM scores, selection bias cannot be excluded, as patients with high disease burden or aggressive para-/extramedullary manifestations may be less likely to proceed to apheresis or infusion. Larger, homogeneous CAR-T and bispecific T-cell engager (BTCE) cohorts will be essential to confirm the applicability of these scoring systems under novel therapeutic conditions. Another limitation is the absence of fat fraction maps in the imaging protocol. While this sequence can support assessment of diffuse infiltration, it is not required for KIM scoring and represents only one of three options in MY-RADS, alongside T1-weighted and high b-value DWI sequences, which were available for all patients. The impact on assessment of diffuse disease should therefore be limited. Additionally, the biological mechanisms underpinning the transition from diffuse to extra- and paramedullary disease remain uncertain. To address these limitations, future research should focus on large-scale, multi-institutional studies incorporating comprehensive molecular analyses including targeted CT-guided biopsies of specific lesions instead of random samples from the iliac crest. Such studies would not only validate the imaging patterns we observed but could also clarify the genetic and microenvironmental drivers of disease evolution in RRMM.

In conclusion, our findings demonstrate that RRMM exhibits distinct imaging patterns characterized by diminished diffuse marrow involvement and increased extra- and paramedullary disease. These results carry substantial implications for MRD diagnostics, prognostication, and therapeutic decision-making. To optimize patient care, comprehensive imaging should be integral to RRMM management, ensuring accurate risk stratification and informed clinical strategies.

## Supplementary information

Supplemental Material

## Data Availability

The datasets generated during and/or analysed during the current study are available from the corresponding author on reasonable request.
