# Supplementary material for: Structured whole-body MRI highlights clinically relevant disease pattern changes in relapsed/refractory multiple myeloma
Source: Leukemia. 2025 Dec 22;40(2):339–47. doi: 10.1038/s41375-025-02834-w (PMC12875875; doi:10.1038/s41375-025-02834-w)
Supplement: Supplementary file 1 — Supplemental Material [file 41375_2025_2834_MOESM1_ESM.pdf]

Supplementary Figures and Tables

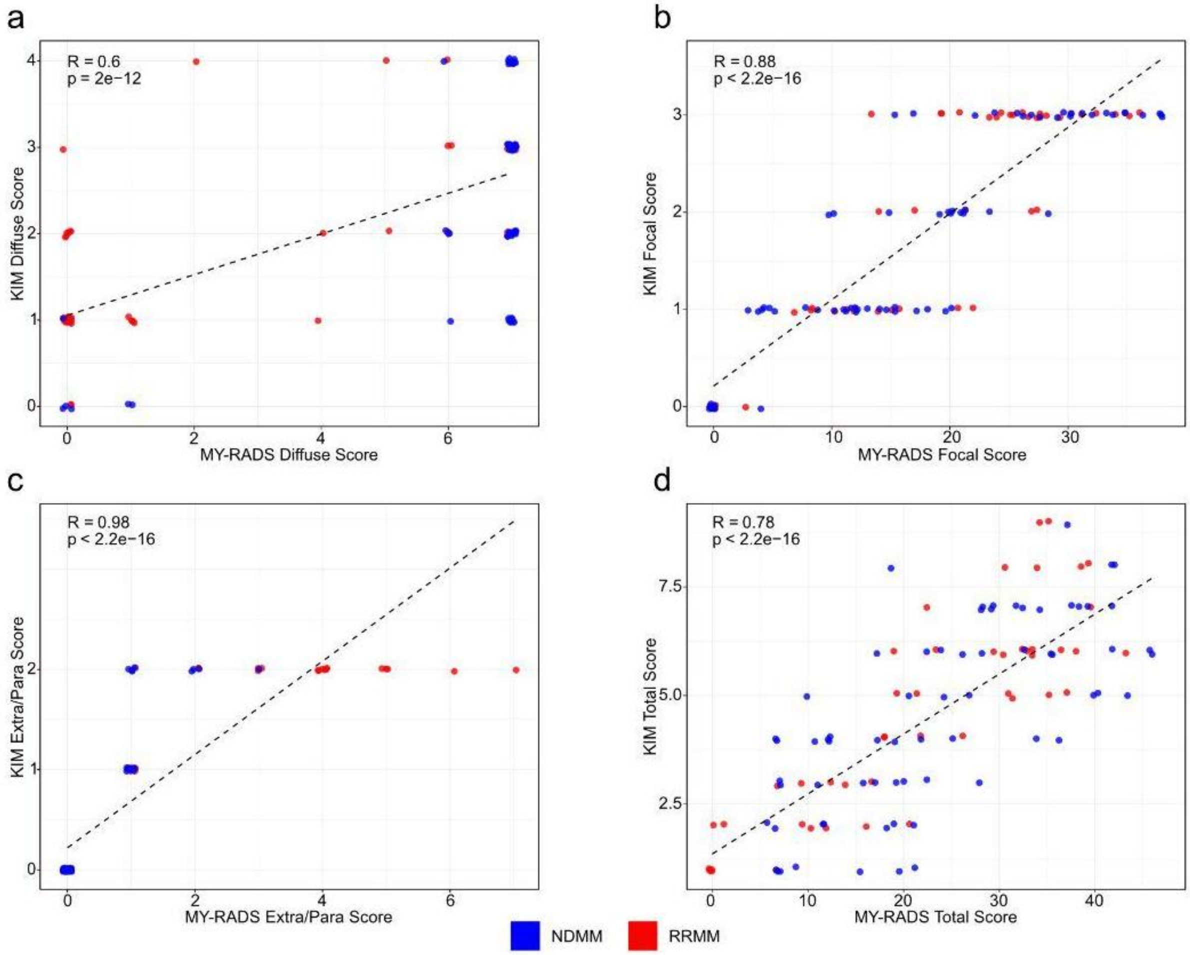

Supplementary Figure 1: Correlation between MY-RADS and KIM scores. Scatter plots show per-patient correlations of MY-RADS and KIM subscores for (a) diffuse infiltration, (b) focal scores, (c) extra-/paramedullary scores, and (d) total score. Blue dots represent NDMM patients, red dots RRMM patients. Pearson correlation coefficients (R) and p-values are indicated in each panel.

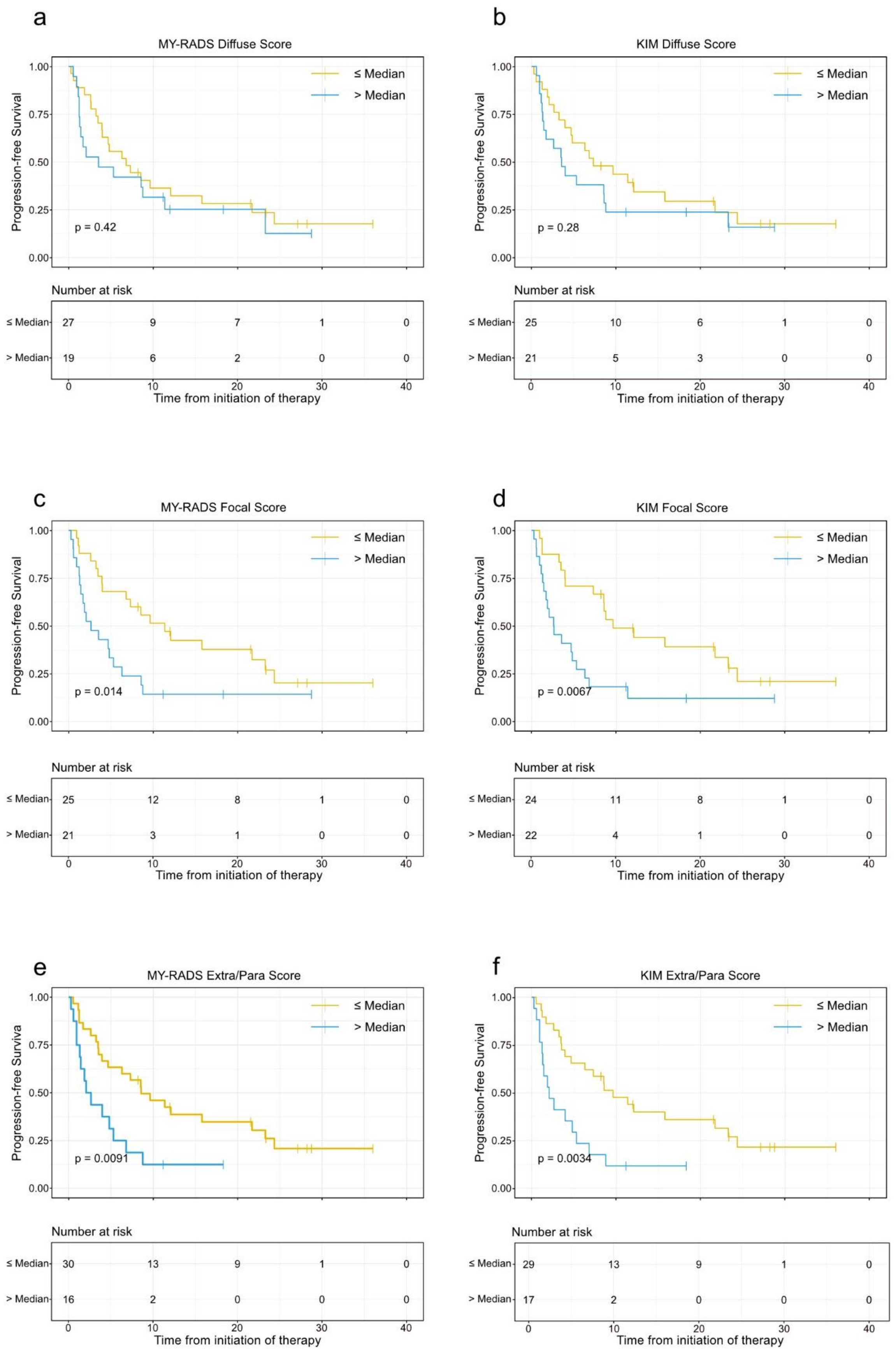
a
MY-RADS Diffuse Score
p = 0.42
b
KIM Diffuse Score
p = 0.28
c
MY-RADS Focal Score
p = 0.014
d
KIM Focal Score
p = 0.0067
e
MY-RADS Extra/Para Score
p = 0.0091
f
KIM Extra/Para Score
p = 0.0034
≤ Median
> Median
Progression-free Survival
Number at risk
Time from initiation of therapy

Supplementary Figure 2: Kaplan-Meier curves for progression-free survival (PFS) in relapsed/refractory multiple myeloma patients, stratified at the median by each subscore of MY-RADS (left panels) and KIM (right panels). **(a,b)** Diffuse subscores; **(c,d)** focal subscores; **(e,f)** extra-/paramedullary subscores. The p-values are from log-rank tests. The tables below each plot show the number of patients at risk over time.

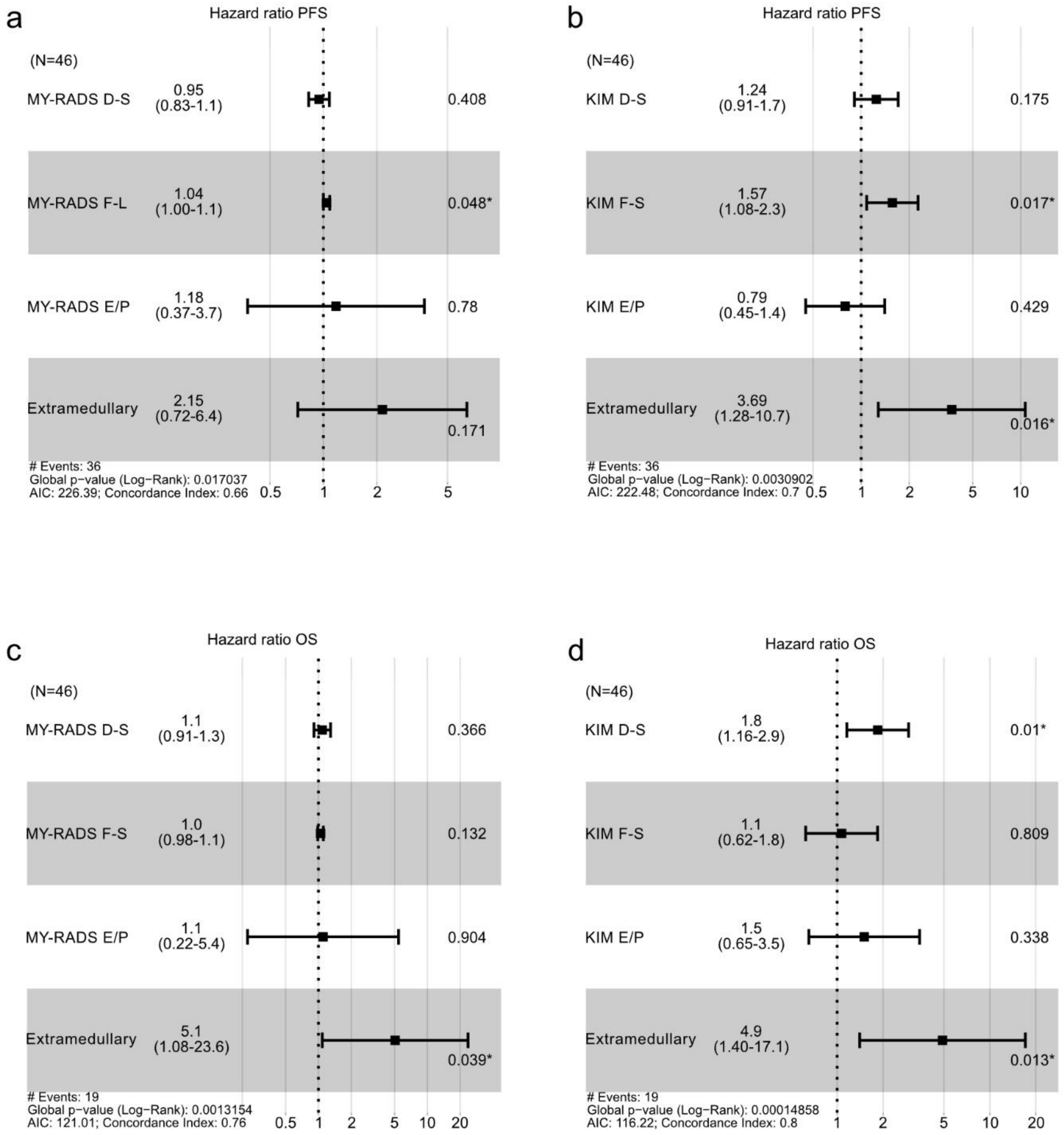

Supplementary Figure 3: Cox proportional hazards models for subscores and EMD of 46 relapsed/refractory myeloma patients. **a)** PFS model including MY-RADS subscores. **b)** PFS model including KIM subscores. **c)** OS model including MY-RADS subscores. **d)** OS model including KIM subscores. D-S: Diffuse Score, F-S: Focal Score, E/P: Extra/Para-Score. Hazard ratios (HR) with 95% confidence intervals appear for each

covariate. The dashed vertical line at HR = 1.0 indicates no effect. Asterisks denote significance (*$p < 0.05$, **$p < 0.01$).

Supplementary Tables

**Treatment**	
**Novel-agent based triplet**	**18**
**Conventional chemotherapy**	**5**
**CAR-T or BTCE**	**14**
**BTCE**	**7**
**Targeted therapy**	**2**

Supplementary Table 1: Treatment Overview

	T1w TSE	T2w STIR	Diffusion-weighted imaging
Sequence	2D TSE	2D TSE STIR	Diffusion-EPI iShim
Plane	coronal	coronal	axial
Slice thickness, distance factor	5.0mm, 10%	5.0mm, 10%	6.0mm, 0%
In-plane resolution (interpolation)	1.3mm x 1.3mm	0.7mm x 0.7mm (interpolated)	1.8mm x 1.8mm (interpolated)
FOV/acquisition matrix	375mmx500mm / 432x768	352mmx470mm / 360x640	287mmx460mm / 160x256
Number of stations (whole-body / pelvis)	5/1	5/1	5/2
Coverage in z-axis per station	375mm	353mm	228mm
Acquisition time per station	1min 43s	1min 7s	2min 49s
Parallel imaging	GRAPPA (Ac.-f. 2)	GRAPPA (Ac.-f. 3)	GRAPPA (Ac.-f. 2)
TR/TE/NSA	528ms/8.4ms/1	3650ms/56ms/1	5130ms/64ms/1
Flip angle	150°	140°	90°
Fat suppression technique	-	slice-selective IR (TI=160ms)	slice-selective IR (TI=180ms)
B-values	-	-	b values: 50/800 s mm^{-2}

Abbreviations: TSE: turbo spin echo; STIR: short-TI inversion recovery; DWI: diffusion-weighted imaging; EPI: echo-planar imaging; iShim: integrated slice-specific dynamic shimming; FOV: field of view; GRAPPA: Generalized Auto-calibrating Partially Parallel Acquisitions; Ac.-f.: acceleration factor. TR: repetition time; TE: echo time; NSA: number of signal averages; IR: inversion recovery, TI: inversion time.

Supplementary Table 2: Imaging parameters

Body region	Score points	Diffuse plasma cell infiltration score (D-S)	Number of focal lesions score (N-S)	Size of the biggest focal lesion score (S-S)	Presence of extra-/ and paramedullary lesions score (E/P-S)	Total score of the body region (Sum of (D-S)+(N-S)+(S-S)+(E/P-S))
Cervical spine	Range	0-1	0-3	0-3	0-1	0-8
	MIN	0	0	0	0	0
	MAX	1	3	3	1	8
Thoracic spine	Range	0-1	0-3	0-3	0-1	0-8
	MIN	0	0	0	0	0
	MAX	1	3	3	1	8
Lumbar spine	Range	0-1	0-3	0-3	0-1	0-8
	MIN	0	0	0	0	0
	MAX	1	3	3	1	8
Pelvis	Range	0-1	0-3	0-3	0-1	0-8
	MIN	0	0	0	0	0
	MAX	1	3	3	1	8
skull	Range	0-1	0-3	0-3	0-1	0-8
	MIN	0	0	0	0	0
	MAX	1	3	3	1	8
Long bones	Range	0-1	0-3	0-3	0-1	0-8
	MIN	0	0	0	0	0
	MAX	1	3	3	1	8
Chest	Range	0-1	0-3	0-3	0-1	0-8
	MIN	0	0	0	0	0
	MAX	1	3	3	1	8
Whole body	Range	0-7	0-21	0-21	0-7	0-56
	MIN	0	0	0	0	0
	MAX	7	21	21	7	56

Legends to Supplementary Table 2: Explanation of the Subscores

Subscore per region	Score point	disease burden
D-S	0	no diffuse disease
	1	diffuse disease
N-S*	0	no focal lesion in the region
	1	1 lesion in the region
	2	2-9 lesions in the region
	3	more than 10 lesions in the region
S-S*	0	no focal lesion
	1	biggest lesion in the region <6mm
	2	biggest lesion in the region 6-15mm
	3	biggest lesion in the region >15mm
E/P-S	0	no extra-/ or paramedullary lesion
	1	extra- or paramedullary lesion

***Legend: According to MY-RADS, two separate subscores for the description of the disease burden adjusted by focal lesions are provided: 1) the subscore for the number of focal lesions (N-S) and 2) the subscore for the maximum size of the focal lesions (S-S). The sum of these two scores collectively describes the focal lesions score (F-S); (N-S + S-S = F-S).**

Supplementary Table 3: MY-RADS analysis scheme for structured reporting of disease burden at BL and FU.

	Mean No-CAR-T Group	Mean CAR-T Group	p-value
Prior lines of therapy	4.97	6.29	0.0688
Age	61.94	60.36	0.256
MY-RADS Diffuse Score	1.47	3.07	0.117
MY-RADS Size Score	11.38	8.57	0.331
MY-RADS Number Score	8.47	6.71	0.26
MY-RADS Focal score	19.84	15.29	0.293
MY-RADS Extra/Para Score	1.91	1.07	0.235
MY-RADS Total score	23.25	19.43	0.519
KIM Diffuse Score	1.66	1.79	0.652
KIM Focal score	2.12	1.43	0.0793
KIM Extra/Para Score	1.06	0.71	0.234
KIM Total score	4.84	3.93	0.223

Supplementary Table 4: Difference in Scores between CAR-T cell treated patients and other patients. P-value was determined using Wilcoxon test.

	No-CAR-T Group (> median)	CAR-T Group (> median)	p-value
MY-RADS Diffuse Score	0.34	0.57	0.26
MY-RADS Focal Score	0.47	0.43	1
MY-RADS Extra/Para Score	0.41	0.21	0.36
MY-RADS Total Score	0.50	0.43	0.90
KIM Diffuse Score	0.44	0.50	0.94
KIM Focal Score	0.53	0.36	0.44
KIM Extra/Para Score	0.44	0.21	0.27
KIM Total Score	0.47	0.29	0.40
Extramedullary	0.28	0.14	0.52

Supplementary Table 5: Distribution of CAR-T cell–treated patients (n=14) compared with other RRMM patients relative to the median values of MY-RADS and KIM subscores. For each score, patients were stratified into two groups (≤ median vs. > median). Proportions for CAR-T and non-CAR-T patients are shown, together with chi-square test p-values.
